# The Influence of Inverted Growth Pattern on Recurrence for Patients with Non-Invasive Low Grade Papillary Urothelial Carcinoma of Bladder

**DOI:** 10.4274/balkanmedj.2017.0081

**Published:** 2017-09-29

**Authors:** Sehbal Arslankoz, İbrahim Kulaç, Dilek Ertoy Baydar

**Affiliations:** 1 Department of Pathology, Hacettepe University School of Medicine, Ankara, Turkey; 2 Clinic of Pathology, Mardin State Hospital, Mardin, Turkey

**Keywords:** urinary bladder, urothelial carcinoma, non-invasive low grade, inverted

## Abstract

**Aims:**

To evaluate the impact of a histologically inverted pattern on recurrence in patients with newly diagnosed non-invasive, low-grade papillary urothelial carcinoma of the urinary bladder.

**Methods:**

A total of 81 patients with primary bladder non-invasive, low-grade papillary urothelial carcinoma diagnosed in a single tertiary-care centre who had at least 1-year follow-up after an initial resection were included. All slides from each case were reviewed to determine the growth pattern (exophytic versus endophytic, i.e. inverted) and other histological parameters. Clinical data were retrieved from hospital records.

**Results:**

Disease recurrence occurred in 41 (50.6%) patients. Cases with an inverted pattern showed a lower recurrence rate than those with pure exophytic tumours (37.5% versus 52.1%), a longer time to first recurrence (mean 34 versus 21.5 months) and fewer recurrence episodes (p=0.482, 0.564 and 0.051, respectively). All recurring inverted cases recurred only once during follow-up. No tumour with >80% inverted architecture recurred.

**Conclusion:**

Our results suggest that non-invasive, low-grade papillary urothelial carcinoma of the bladder tends to have a better outcome in terms of disease recurrence if it shows an inverted growth pattern. To indicate the presence and percentage of the inverted pattern in low-grade urothelial carcinomas in the pathology report might be considered as an adjunct to help long-term patient management.

Non-invasive, non-papilloma bladder tumours represent the majority (70%) of bladder neoplasms (1). Although their progression rate is low, they are characterized by frequent relapses. The estimated risk of recurrence during the first year after diagnosis is 15%-70% (2,3,4). Urothelial cancers with an inverted proliferation pattern have been thought to be similar to exophytic papillary cancer with regard to grading, multiplicity, invasiveness and recurrence despite the lack of evidence-based data. In this study, we investigate the relevance of an inverted tumour growth pattern for low grade bladder urothelial carcinoma recurrence after initial resection.

## MATERIALS AND METHODS

All bladder transurethral resection (TUR) cases between 2000 and 2011 with an initial (de novo) diagnosis of low-grade, non-invasive papillary urothelial carcinoma were identified in the electronic patient data system of a single tertiary care university hospital. Individuals with previous or concomitant upper urinary tract tumours or diagnosis of urothelial carcinoma in situ were excluded. Among the remaining cases, patients with at least one-year follow-up in the same institution (n=81) formed the cohort of the study.

Clinical information regarding patients' age, gender, tumour size, location, focality and follow-up data were obtained from hospital files or electronic records. Application of intravesical treatment (IVT) with bacillus Calmette-Guérin or intravesical chemotherapy was noted for each patient. Their pathology slides were retrieved from the archives and re-evaluated to note the histological parameters, namely growth pattern (exophytic versus inverted), presence and extent of necrosis, apoptosis, inflammatory infiltrate, nucleolar prominence and mitotic rate as well as for tumour typing and grading according to the 2016 WHO/ISUP classification (5). Eight tumours were observed to reveal an inverted pattern to a varying degree. The amount of inverted growth was estimated as a percentage of the total tumour. Recurrence was defined as disease reappearance occurring at least 3 months after the initial diagnosis. The study was approved by the institutional ethics review board.

### Statistical analysis

Mean, median and minimum-maximum values of numeric parameters were analysed. Chi-square, McNemar and Mann-Whitney U tests were used for categorical and continuous variables. A non-parametric Spearman correlation coefficient was used where needed. Results were considered statistically significant at p<0.05. All statistical analysis was performed using the IBM SPSS Statistics (Version 15.0) software package.

## RESULTS

### Patient demographics and clinical features

Males formed 80% of our study group (65 males and 16 females). Mean length of follow-up was 84 months (median 80, range 15-319 months), and mean patient age at diagnosis was 61 years (36-86). Mean tumour size was 2.3 cm (0.2-8 cm), and 29 tumours (42%) were multifocal. During disease surveillance, 18 of the patients with recurrent tumours were given IVT.

### Characteristics of cases with the inverted pattern

**FIG. 1.**
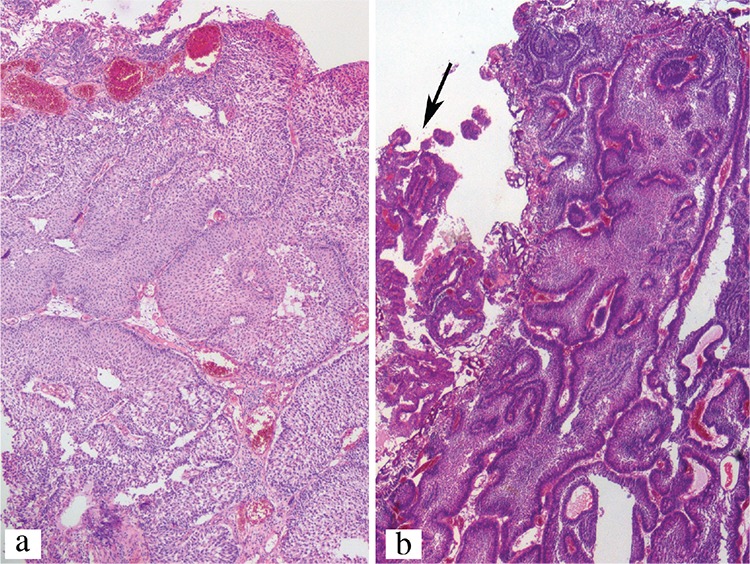
Two examples of non-invasive, low-grade papillary urothelial carcinoma with an inverted pattern, Case 3 (H&E x40) (a), Case 4. Accompanying small exophytic component is visible in the left upper part of the image indicated by an arrow (b) (H&E x40)

The inverted pattern was found in eight patients (9.9%), who had a median age of 54 years (mean 57; range, 42-84 years) and a male to female ratio of 7:1 (Table 1). The percentage of the inverted component varied from 40% to 100%, with 4 cases being >80% (Figure 1a, 1b). Their mean length of follow-up was 82.1 months (36-128 months). The mean tumour size was 26.7 mm (6-60 mm). Two of the eight cases had multifocal tumours.

As summarized in Table 2, when the two groups (cases with and without the inverted pattern) were compared with respect to patient age, gender, history of smoking, tumour size, tumour focality, IVT administration and duration of follow-up, no statistically significant difference was found (all p>0.05).

### Recurrence

Bladder tumour recurrence was documented in 41 patients (50.6%). The mean time to recurrence was 22 months (2-86 months). Large tumour size was related to the risk of recurrence (p=0.032). Mean tumour diameter was 27.82 mm (median 25 mm) in cases that showed recurrence during follow-up, whereas this figure was 19.03 mm (median 20 mm) in non-recurrent lesions. There were no significant differences in age, gender or smoking status between the recurrent and non-recurrent groups.

Three out of eight tumours (37.5%) with an inverted pattern showed recurrence during their subsequent biopsies. The recurrence rate was 38 (52.1%) for cases with pure exophytic lesions (p=0.482). It was noted that none of the four cases with more than 80% inverted component recurred.

The mean time to first recurrence for the cases with an inverted growth pattern was 34 months (median 9, range 7-86 months). This was 21.5 months (median 13.5, range 2-80 months) for cases with pure exophytic pattern tumours (p=0.564).

All recurrent cases in the inverted pattern group had only one recurrence episode during their follow-up, whereas the mean number of recurrence episodes per case was 3.11 (median 2, range 1-14) for the pure exophytic tumours (p=0.051). Recurrences of 10 cases out of 81 (12.3%) showed either stage and/or grade progression during their follow-up. These belonged to the pure exophytic group (stage progression in 2, grade progression in 6, and both stage and grade progression in 2 cases). There was no progression observed in the recurrences of the tumours with the endophytic pattern (p=0.587).

## DISCUSSION

Bladder cancer represents a wide range of tumour biology and behaviours. The difficulty in envisioning tumour behaviour and prognosis highlights the need to further explore the predictive factors. Stage progression and cancer death are rare in non-invasive, low-grade papillary urothelial carcinoma (LGUC). On the other hand, tumour recurrence is a common problem that occurs in up to 70% of patients (6). This promotes cautious surveillance with cystoscopy and life-long follow-up for patients with non-invasive bladder cancer (7,8,9). Although there are some attempts, no reliable (and definite) set of parameters has yet been identified to determine the risk of recurrence in these tumours. The suggested indicators of a high potential for recurrence in a low-grade disease are male gender, large tumour size, increased number of tumour foci and having a rate of >1 recurrence/year (4,6,10). The adverse influence of tumour size on recurrence has been demonstrated in our study similarly to other series (11,12,13). Primary tumour size was a predictor of intravesical recurrence following TUR, as large tumours are more likely to recur than smaller ones. Studies on the recognition of additional prognostic factors that will provide an improved approach for postoperative monitoring and surveillance and potentially improve patient outcomes are still extremely needed in urothelial carcinomas.

Urothelial neoplasia may show variable histologies. Most papillary urothelial carcinomas are characterized architecturally by an exophytic growth of finger-like papillae, but some exhibit a prominent inverted, in other words, endophytic growth pattern, which may sometimes create considerable difficulty for the pathologist in assessing invasion histologically. The inverted growth pattern can be associated with any form of urothelial neoplasia, including papilloma, urothelial neoplasm of low malignant potential and low and high grade urothelial carcinomas, which can be non-invasive or invasive. Anastomosing cords and columns of urothelium beneath the surface epithelium or broad pushing bulbous invaginations into the lamina propria are seen microscopically. The effect of the presence and extent of inverted pattern on the recurrence rate of urothelial malignancies is largely unknown.

The only study that looks at the biologic behaviour of inverted urothelial lesions other than papilloma is the one by Maxwell et al. (14), which included 186 primary papillary urothelial neoplasias of low malignant potential (PUNLMP), out of which 12 had an exclusively inverted growth pattern. The authors found no recurrence or progression in their 12 inverted PUNLMPs, whereas overall rates of recurrence and progression to high grade carcinoma in their series were 20.1% and 1.6%, respectively, in a mean follow-up of 61 months.

The impact of inverted growth in non-invasive bladder cancer has not been investigated thus far. Our study focuses on low-grade, non-invasive urothelial carcinoma with a total or partially inverted pattern. We have reviewed 81 patients with primary, LGUC and evaluated whether histologically inverted growth has been associated with tumour recurrence. The prevalence of an inverted component in the first diagnostic bladder biopsy among LGUC cases was 9.9% in our series. They had similar demographical and pathological features to their exophytic counterparts. However, lesions with an inverted element revealed a less problematic clinical course, as they had a lower rate of recurrence than non-inverted tumours: 37.5% versus 52.1%. Additionally, the mean duration to first recurrence was approximately 1 year longer in cases with an endophytic component: 34 months in contrast to 21.5 months for classic papillary carcinomas. Despite the data clearly indicating a tendency towards a better clinical course when tumours harbour any endophytic growth pattern, the differences did not reach statistical significance (p=0.482 and p=0.564 respectively), which we believe was due to the low patient number. More importantly, we found an obvious distinction in the overall number of total recurrences between the two groups. Three out of our eight inverted LGUCs recurred, and they recurred only once during their follow-up in contrast to the mean number of recurrence episodes of 3.11 for the pure exophytic tumours. The statistical test showed that this difference was borderline significant with a p value of 0.051 and was most probably a reflection of the small cohort size. Additional studies including longer intervals for surveillance cystoscopy for the patients with inverted LGUC may be needed to verify the present findings. It is worth emphasizing that none of our four cases with inverted components of more than 80% have recurred.

In conclusion, our results suggest that having any percentage of inverted pattern in a LGUC of the bladder may imply a low recurrence risk. This is especially true when the inverted pattern is exclusive in the lesion. Unfortunately, the limited number of patients in the current study precludes a definitive conclusion with statistical strength. Larger prospective studies are required to better characterize the biologic behaviour of inverted growth in LGUC.

## Figures and Tables

**Table 1:**
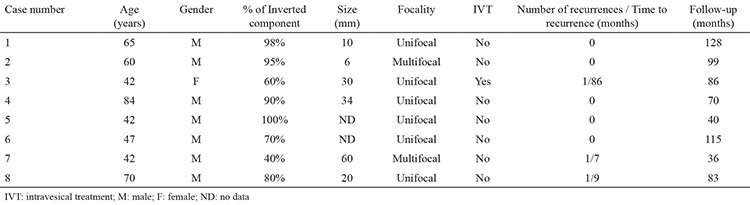
Clinical features and tumour characteristics of eight cases with an inverted pattern

**Table 2:**
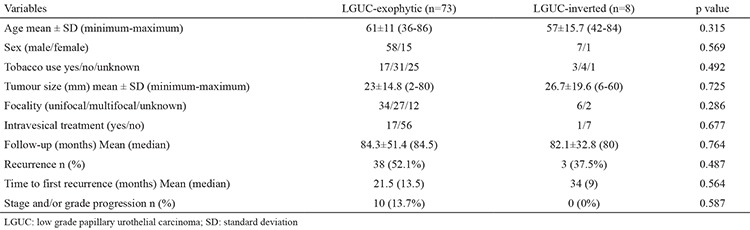
Comparison of some clinical features between exophytic and inverted LGUC
